# Sequential Assembly of Centromeric Proteins in Male Mouse Meiosis

**DOI:** 10.1371/journal.pgen.1000417

**Published:** 2009-03-13

**Authors:** María Teresa Parra, Rocío Gómez, Alberto Viera, Elena Llano, Alberto M. Pendás, Julio S. Rufas, José A. Suja

**Affiliations:** 1 Unidad de Biología Celular, Departamento de Biología, Edificio de Biológicas, Facultad de Ciencias, Universidad Autónoma de Madrid, Madrid, Spain; 2 Instituto de Biología Molecular y Celular del Cáncer (CSIC-USAL), Campus Miguel de Unamuno, Salamanca, Spain; 3 Departamento de Fisiología, Campus Miguel de Unamuno, Salamanca, Spain; The University of North Carolina at Chapel Hill, United States of America

## Abstract

The assembly of the mitotic centromere has been extensively studied in recent years, revealing the sequence and regulation of protein loading to this chromosome domain. However, few studies have analyzed centromere assembly during mammalian meiosis. This study specifically targets this approach on mouse spermatocytes. We have found that during prophase I, the proteins of the chromosomal passenger complex Borealin, INCENP, and Aurora-B load sequentially to the inner centromere before Shugoshin 2 and MCAK. The last proteins to be assembled are the outer kinetochore proteins BubR1 and CENP-E. All these proteins are not detected at the centromere during anaphase/telophase I and are then reloaded during interkinesis. The loading sequence of the analyzed proteins is similar during prophase I and interkinesis. These findings demonstrate that the interkinesis stage, regularly overlooked, is essential for centromere and kinetochore maturation and reorganization previous to the second meiotic division. We also demonstrate that Shugoshin 2 is necessary for the loading of MCAK at the inner centromere, but is dispensable for the loading of the outer kinetochore proteins BubR1 and CENP-E.

## Author Summary

The centromere is a chromosome domain essential for the correct partitioning of chromosomes during mitotic and meiotic cell divisions. The characterization of the centromeric proteins and their sequential assembly have been extensively studied in mammalian mitosis, since defective chromosome segregation is associated with birth defects and cancer. However, few studies have analyzed the centromere assembly during meiosis, a special cell division leading to the production of haploid gametes. Here, we analyze the sequence of loading of several centromeric and kinetochoric proteins during male mouse meiosis. We show that during both meiotic divisions, the proteins of the chromosomal passenger complex Borealin, INCENP, and Aurora-B load sequentially to the inner centromere before Shugoshin 2 and MCAK. The outer kinetochore proteins BubR1 and CENP-E are the last ones to be assembled. We also demonstrate, using a knockout mouse for *Sgol2*, that the inner centromeric protein Shugoshin 2 is dispensable for the loading of the outer kinetochore proteins BubR1 and CENP-E, but necessary for the assembly of MCAK. This study shows that the analysis of the behavior of different centromere proteins during meiosis can offer new insights concerning centromere organization.

## Introduction

Accurate chromosome segregation in mitosis is crucial to maintain a diploid chromosome number. Errors in chromosome segregation result in aneuploid daughter cells which are prone to become malignant. The centromere is the chromosome domain that directs this segregation process since it is involved in relevant events such as sister-chromatid cohesion, the spindle assembly checkpoint (SAC), the attachment to spindle microtubules (MTs) and chromosome movements [1]–[4].

The centromere is structurally divided into the kinetochore and the inner centromere domains. The kinetochore is a proteinaceus structure at the centromere surface mostly involved in the attachment of spindle MTs, chromosome movements and SAC regulation [2],[3],[5]. In vertebrates, this domain is subdivided into three distinct regions: the inner, the central and the outer kinetochore plates [2],[3],[6]. The inner kinetochore plate is formed by the chromatin subjacent to the kinetochore, in which histone H3 is replaced by CENP-A [7],[8], and additional constitutive proteins that appear at kinetochores throughout the cell cycle. On the other hand, the outer kinetochore plate and the fibrous corona, detected only in prometaphase, are mainly composed of MT motor proteins, such as CENP-E and cytoplasmic dynein, as well as SAC proteins, as for instance Bub1, BubR1, Mad1 and Mad2 [2],[3],[5],[6].

The inner centromere domain is the region spanning between sister kinetochores. Several proteins with different functions have been localized in this region [9]. Some proteins like CENP-B are constitutive, while many others are incorporated to the inner centromere at specific cell cycle stages. This is the case for the chromosomal passenger complex (CPC) proteins INCENP, the kinase Aurora-B, Survivin and Borealin/Dasra. The CPC has been involved in many different functions such as chromatin modifications (through the phosphorylation of histone H3), correction of kinetochore attachment errors, the SAC, the assembly of a stable bipolar spindle, and completion of cytokinesis [9],[10]. Another group of inner centromere proteins are the cohesin complexes that maintain sister chromatids tightly associated until their segregation in anaphase [11],[12]. Cohesin complexes are located between sister chromatids along their entire length, but interestingly, during mitosis most of them are released from chromosome arms during prophase/prometaphase [13], while the centromeric ones are protected until the metaphase/anaphase transition [14]. It has been proposed that two proteins placed at the inner centromere, shugoshins SGOL1 and/or SGOL2, protect centromeric cohesin complexes from cleavage by separase until the onset of anaphase [15],[16]. Additionally, the MT depolymerizing kinesin MCAK is also found at this domain [17]–[19] and is involved in the correction of improper kinetochore-MTs attachments [17],[20].

The precise sequence of loading of proteins to the centromere and the kinetochore is largely unknown. While constitutive proteins of the inner kinetochore are present all along the cell cycle, some of the inner centromere proteins are loaded during prophase and most outer kinetochore proteins are assembled during prometaphase after nuclear envelope breakdown. Thereafter, most of these proteins are released from the centromere and the kinetochore after the inactivation of the SAC during anaphase [21],[22]. CENP-A has a crucial role in centromere specification and kinetochore assembly since most kinetochore proteins need, either directly or indirectly, its presence to be properly incorporated to the kinetochore [3],[22],[23]. In this context, CENP-A is needed for the incorporation of the constitutive inner kinetochore proteins CENP-C, -H and -I, at least in mouse and *Caenorhabditis elegans* [24]. These constitutive proteins are in turn needed for the loading of the outer kinetochore proteins CENP-E, CENP-F and SAC proteins [22],[24],[25]. These outer kinetochore proteins also present a precise loading sequence. For instance, Bub1 and BubR1 are necessary for the correct loading and function of CENP-E and other SAC proteins [26],[27].

Kinetochore proteins are also needed to recruit proteins to the inner centromere. There is an ongoing controversy over whether CENP-A is required for the localization of Aurora-B. While some studies support that Aurora-B needs CENP-A for its loading [28]–[30], other reports suggest that this loading is independent of CENP-A [31]. CENP-A also seems to condition the localization of MCAK [30]. Additionally, Bub1 is necessary for the accurate localization of MCAK [30], the stability and correct positioning of CPC, and the binding of Shugoshin to the inner centromere [32]. Regarding the CPC, different studies have revealed that Borealin, INCENP, Aurora-B and Survivin form a complex in which each subunit seems to be necessary for the loading of the others [9], and the immunodepletion of any of them caused the removal of the others [33]. Borealin might be the key protein, since it can bind DNA *in vitro* [31] and Dasra-A, the Borealin-related protein in *Xenopus*, is necessary for the loading of the other CPC proteins [34]. However, other proteins like INCENP, which can bind the histone variant H2Az [35], or Aurora-B, which may be recruited at the centromere through the phosphorylation of CENP-A by Aurora-A [28], may be also important for CPC loading. Alternatively, it has also been suggested that SGOL2 is needed for the correct loading of the CPC proteins in yeast since, in its absence, Aurora-B can not be targeted to the centromeres [36],[37]. Moreover, SGOL2 is needed for the loading of MCAK at centromeres in human cells [38].

The diverse pathways leading to the recruitment of outer kinetochore and inner centromere proteins have been mainly studied in mitotic chromosomes, whereas little information is available regarding the assembly of centromeres and kinetochores during mammalian meiosis. Previous reports have shown that in male mouse meiosis, INCENP is loaded at the pericentromeric chromatin before Aurora-B [39], and that MCAK is loaded after Aurora-B [19]. In this study, we have analyzed the loading sequence of the inner centromere proteins Borealin, INCENP, Aurora-B, SGOL2, and MCAK, and the outer kinetochore proteins CENP-E and BubR1, during both male mouse meiotic divisions. Additionally, we have used a knockout mouse for *Sgol2* to analyze the influence of this protein in the loading of MCAK and the outer kinetochore proteins CENP-E and BubR1. Our results lead us to present a working model for the sequential assembly of centromere and kinetochore proteins during meiosis.

## Results

### Sequential Loading of CPC Proteins

The constitutive kinetochore proteins revealed by an anti-centromere autoantibody are located at kinetochores from the beginning of meiosis [40]. However, most of the inner centromere and outer kinetochore proteins are loaded at different times during both meiotic divisions. In order to delineate the loading sequence of the CPC proteins Borealin, INCENP, and Aurora-B we made double immunolabelings on spermatocytes. Unfortunately, we were unable to detect Survivin even though we used several antibodies.

The double immunolabeling of INCENP and SYCP3, a structural component of synaptonemal complex lateral elements, allowed us to determine previously that INCENP labels the synaptonemal complex central element from zygotene up to mid/late pachytene when it begins to relocalize to heterochromatic chromocenters, while Aurora-B appears at chromocenters later in diplotene [39]. In this study we compared the relative loading of these two proteins with Borealin.

**Figure 1:**
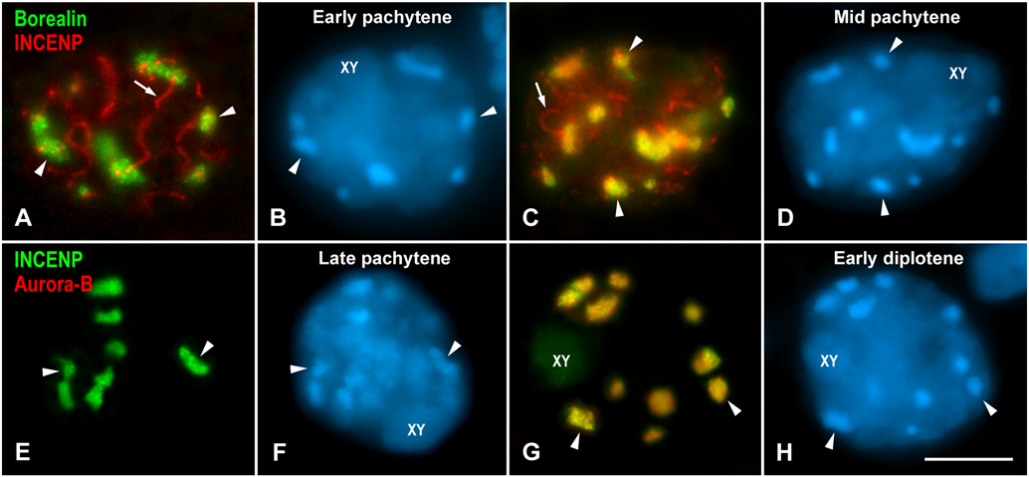
Loading of CPC proteins in prophase I spermatocytes. (A–D) Early and mid pachytene spermatocytes double immunolabeled for Borealin (green) and INCENP (red). INCENP appears at synaptonemal complex stretches (arrows). Both proteins colocalize at chromocenters (arrowheads) only in mid pachytene. (E–H) Late pachytene and early diplotene spermatocytes double immunolabeled for INCENP (green) and Aurora-B (red). Both proteins colocalize at chromocenters (arrowheads) in early diplotene. The spermatocytes shown are projections of several focal planes, and are counterstained with DAPI (blue). The sex body is indicated (XY). Scale bar 10 µm.

We found that Borealin appeared at chromocenters during pachytene when INCENP was still only present at synaptonemal complexes (Figure 1A and 1B). The chromocenters represent clustered centromere heterochromatic regions that are clearly discerned after DAPI staining, and located at the nuclear periphery. However, since we have projected different focal planes through the spermatocytes, some chromocenters appear in the middle of the nuclei (Figure 1A and 1B). In other pachytene spermatocytes, Borealin and INCENP colocalized at chromocenters whereas INCENP was also visualized at synaptonemal complexes (Figure 1C and 1D). Taking into account these results we considered that Borealin first appeared at early pachytene, while INCENP began to redistribute from synaptonemal complexes to chromocenters in mid pachytene. These proteins colocalized at centromeres from mid pachytene up to late anaphase I. The labeling of INCENP at synaptonemal complexes became undetectable at late pachytene (Figure 1E and 1F) as previously reported [39]. Although INCENP was present at chromocenters in late pachytene, Aurora-B was not detected at this stage (Figure 1E and 1F). Aurora-B became first detectable at chromocenters later, during early diplotene, colocalizing with INCENP (Figure 1G and 1H). From diplotene onwards, the three CPC proteins colocalized.

### Loading of the Inner Centromere Proteins SGOL2 and MCAK

**Figure 2:**
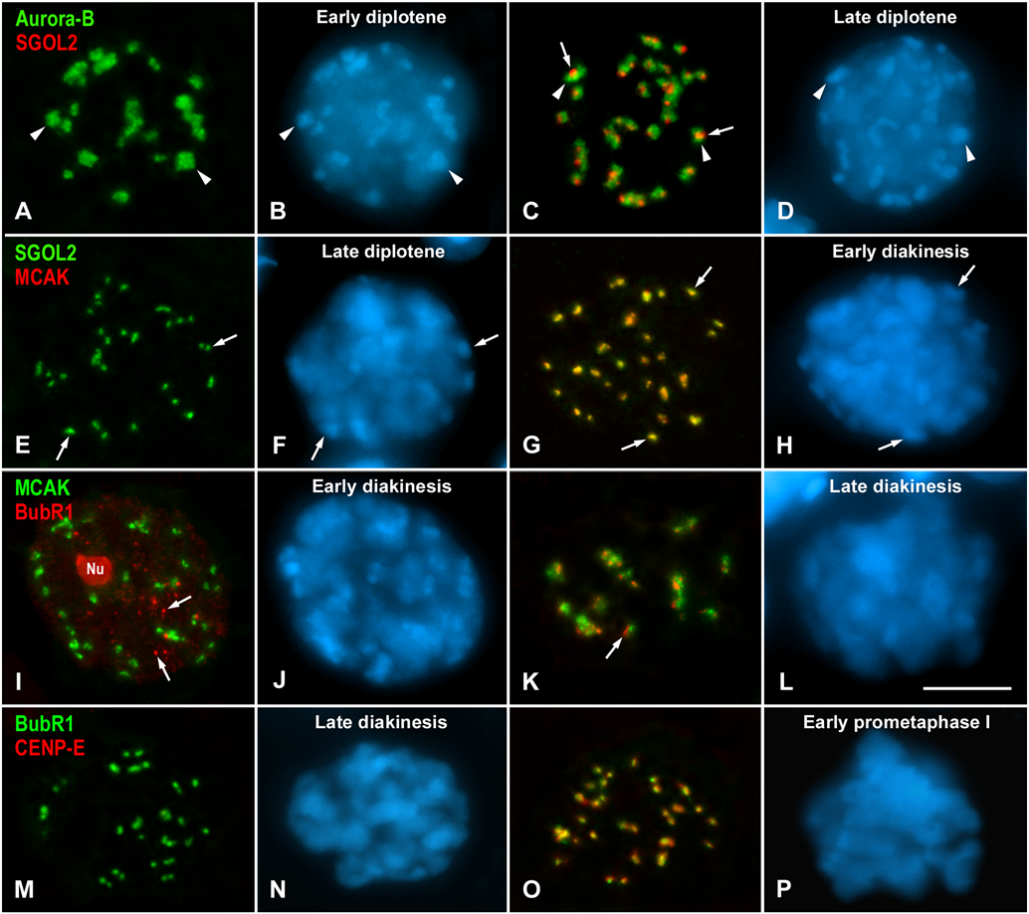
Loading of SGOL2, MCAK, BubR1 and CENP-E. (A–D) Early and late diplotene spermatocytes double immunolabeled for Aurora-B (green) and SGOL2 (red). SGOL2 first appears in late diplotene as discrete signals (arrows) at chromocenters (arrowheads). (E–H) Late diplotene and early diakinesis spermatocytes double immunolabeled for SGOL2 (green) and MCAK (red). MCAK colocalizes with SGOL2 from early diakinesis on at the centromeric regions (arrows) as revealed by the DAPI labelling. (I–L) Early and late diakinesis spermatocytes double immunolabeled for MCAK (green) and BubR1 (red). In early diakinesis, BubR1 is present at the residual nucleolus (Nu) and at small nucleoplasmic aggregates (arrows). BubR1 is first detected as small signals (arrow) adjacent to the MCAK-labeled centromeres in late diakinesis. (M–P) Late diakinesis and early prometaphase I spermatocytes double immunolabeled for BubR1 (green) and CENP-E (red). CENP-E loads to the kinetochores by early prometaphase I, where the condensed bivalents start to be discerned with the DAPI staining. All spermatocytes shown are projections of several focal planes, and are counterstained with DAPI (blue). Scale bar 10 µm.

**Figure 3:**
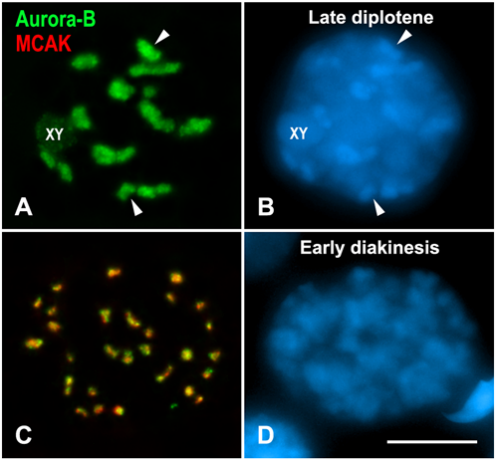
Aurora-B and MCAK labeling in late diplotene (A, B) and early diakinesis (C, D) spermatocytes. Spermatocytes are double immunolabeled for Aurora-B (green) and MCAK (red), and counterstained with DAPI (blue). The Aurora-B signals at chromocenters (arrowheads) concentrate with ongoing chromosome condensation from late diplotene up to early interkinesis when it then colocalizes with MCAK. The sex body is indicated (XY). Scale bar 10 µm.

We next studied the timing of centromere loading of SGOL2 and MCAK, which are present at the inner centromere in metaphase I [19],[41]. We have previously analyzed the time of appearance of SGOL2 at centromeres by double immunolabeling with the cohesin subunit RAD21, which labels cohesin axes that are coincident with the synaptonemal complex lateral elements, and can then be used to accurately stage prophase I spermatocytes [42]. Likewise, we have already analyzed the loading time of MCAK by double immunolabeling with SYCP3. These studies showed that SGOL2 and MCAK were loaded at centromeres by late diplotene [19],[41]. However, we did not know the relative loading sequence of these two proteins. Since we had found that Aurora-B was the last CPC protein loaded at centromeres, we used it as a marker to ascertain the loading time of SGOL2 and MCAK. During early diplotene, when Aurora-B labeled the chromocenters, no labeling was found for SGOL2 (Figure 2A and 2B) or MCAK (data not shown). However, SGOL2 became detectable at centromeres later, by late diplotene, as dotted signals close to or inside the Aurora-B labeled chromocenters (Figure 2C and 2D). On the other hand, when spermatocytes where double immunolabeled for SGOL2 and MCAK, some of them only showed SGOL2 labeling (Figure 2E and 2F), while in other ones both proteins colocalized at centromeres (Figure 2G and 2H). Thus, SGOL2 is loaded at centromeres during late diplotene, and MCAK loads later at very late diplotene or in early diakinesis. The location of SGOL2 and MCAK was identical, indicating that these two proteins colocalized at the inner centromere. Interestingly, at the time SGOL2 and MCAK were loaded to the centromere, the CPC proteins, which in previous stages occupied the entire chromocenters, had changed their distribution. Thus, from late diplotene up to early diakinesis, and concomitantly with ongoing chromosome condensation, the CPC proteins appeared as more discrete signals that colocalized with MCAK and SGOL2 (Figure 3).

### Loading of the Outer Kinetochore Proteins CENP-E and BubR1

We also analyzed the loading of the outer kinetochore proteins CENP-E and BubR1 [43],[44]. Taking into account bivalent condensation, we had reported that during male mouse meiosis CENP-E was first detectable at kinetochores during late diakinesis/early prometaphase I [39],[40]. However, there are not data about the relative loading sequence of CENP-E and BubR1. These proteins were first found at kinetochores once the CPC proteins and SGOL2 and MCAK had been recruited to the inner centromere. In early diakinesis spermatocytes, when MCAK was already loaded at the inner centromere, neither CENP-E nor BubR1 could be detected at kinetochores (Figure 2I and 2J).

During zygotene and pachytene, BubR1 was detected as large nucleoplasmic masses (Figure S1A–S1D). A double immunolabeling of BubR1 and fibrillarin, a nucleolar protein, demonstrated that the BubR1 nuclear masses corresponded to nucleoli lying in the nucleoplasm or associated to the sex body (Figure S2A and S2B). From diplotene up to early diakinesis, BubR1 was visualized at the disintegrating nucleoli and numerous smaller aggregates in the nucleoplasm (Figures S1E–S1H and S2C and S2D). However, these smaller BubR1 aggregates did not colocalize with either the kinetochores, as revealed by an ACA serum (Figure S1E–S1H), or the inner centromere protein MCAK (Figure 2I and 2J). BubR1 was first detected onto kinetochores at late diakinesis. During this stage, identified by the absence of a sex body typical of the pachytene and diplotene stages, and showing condensed bivalents, BubR1 was no longer detected at small nucleoplasmic aggregates (Figure S1J and S1K). BubR1 appeared as plates or dots near the larger MCAK signals (Figure 2K and 2L). Following BubR1 incorporation, CENP-E became loaded to kinetochores. CENP-E was not detectable at late diakinesis (Figure 2M and 2N), but was clearly found later at early prometaphase I kinetochores colocalizing with BubR1 after nuclear envelope breakdown (Figure 2O and 2P).

### Loading of MCAK, BubR1, and CENP-E in the Absence of SGOL2

**Figure 4:**
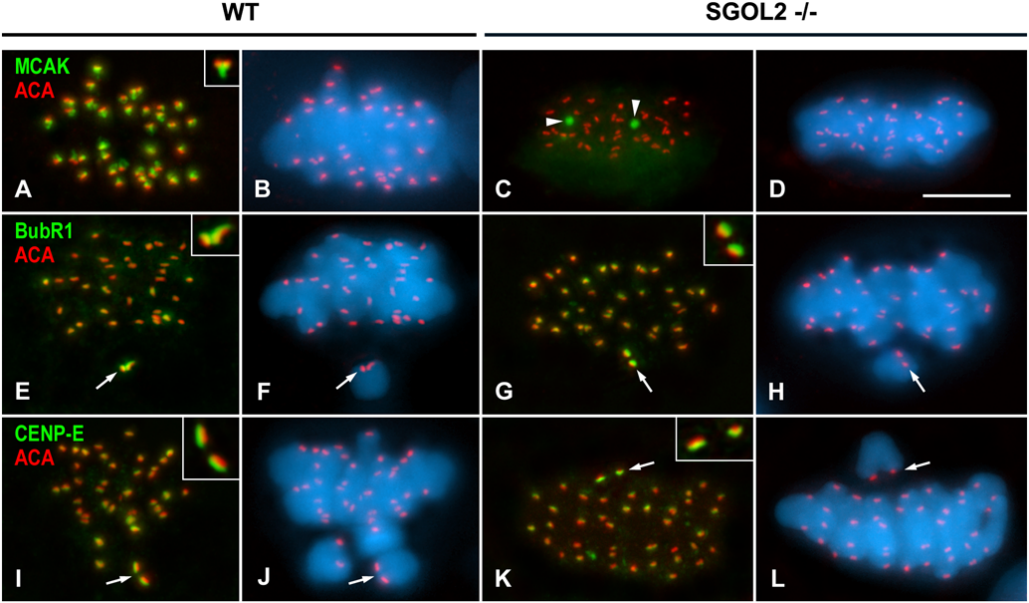
Distributions of MCAK, BubR1 and CENP-E in wild-type and *Sgol2* ^−/−^ prometaphase I and metaphase I spermatocytes. (A–D) Metaphase I spermatocytes double immunolabeled for MCAK (green) and kinetochores (ACA, red). MCAK is accurately located at the inner centromere domain below the closely associated sister kinetochores (inset) in wild-type (WT) spermatocytes, but delocalized as one or two cytoplasmic aggregates (arrowheads) in *Sgol2* ^−/−^ spermatocytes. (E–H) Prometaphase I spermatocytes double immunolabeled for BubR1 (green) and kinetochores (ACA, red). In both wild-type and *Sgol2* ^−/−^ spermatocytes, BubR1 preferentially labels the kinetochores (arrows) of unaligned bivalents. (I–L) Prometaphase I spermatocytes double immunolabeled for CENP-E (green) and kinetochores (ACA, red). CENP-E appears enriched at kinetochores (arrows) of unaligned bivalents. Both BubR1 and CENP-E appear at the outer kinetochore above the ACA signals (insets in E–L). All spermatocytes shown are projections of several focal planes, and are counterstained with DAPI (blue). Scale bar 10 µm.

A recent study proposes that SGOL2 is needed for the loading of MCAK at the inner centromere of mitotic chromosomes [38]. We then analyzed whether MCAK, as well as BubR1 and CENP-E, were loaded at the inner centromere and outer kinetochore, respectively, in the absence of SGOL2 in male knockout mice for *Sgol2* [45]. Our results showed that MCAK, that is found at the inner domain of wild-type metaphase I centromeres (Figure 4A and 4B) [19], was not present at centromeres in metaphase I *Sgol2^−/−^* spermatocytes. Instead, MCAK only appeared at one or two round cytoplasmic aggregates (Figure 4C and 4D). By contrast, BubR1 (Figure 4E–4H) and CENP-E (Figure 4I–4L) loaded accurately to be present at the outer kinetochore in metaphase I *Sgol2^−/−^* spermatocytes. Moreover, these two proteins, that are involved in the regulation of the SAC, appeared enriched at kinetochores of unaligned bivalents in both wild-type (Figure 4E, 4F, 4I, and 4J) and *Sgol2^−/−^* (Figure 4G, 4H, 4K, and 4L) metaphase I spermatocytes.

### Reloading of Centromeric Proteins during the Second Meiotic Division

During male mouse meiosis, the CPC proteins INCENP and Aurora-B, and also CENP-E, relocalize from the centromeres to the spindle midzone during late anaphase I [39], whereas SGOL2 and MCAK disappear from centromeres during the telophase I/early interkinesis transition [19],[41], and BubR1 is lost from kinetochores during anaphase I/telophase I (Figure S1M–S1T). Accordingly, all these proteins were not present at the centromeres in early interkinesis nuclei and need to be reloaded to the centromere in preparation for the second meiotic division.

**Figure 5:**
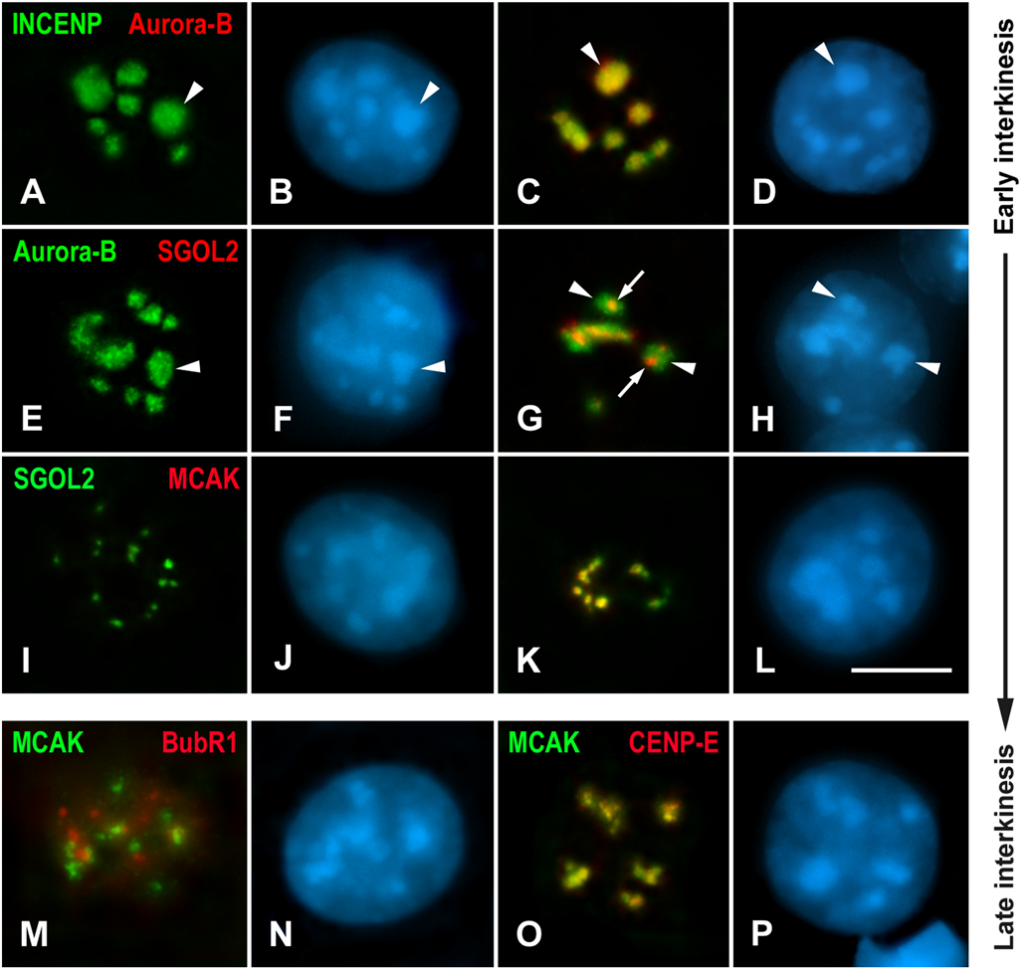
Loading of centromeric proteins throughout interkinesis. (A–D) Nuclei are double immunolabeled for INCENP (green) and Aurora-B (red). All nuclei show INCENP at chromocenters (arrowheads), but only in some of them Aurora-B colocalizes with INCENP. (E–H) Nuclei double immunolabeled for Aurora-B (green) and SGOL2 (red). Among all the nuclei where Aurora-B appears at chromocenters (arrowheads) only some of them show SGOL2 as small signals (arrows) inside chromocenters. (I–L) Nuclei double immunolabeled for SGOL2 (green) and MCAK (red). MCAK appears as small signals inside the chromocenters after, and colocalizing with SGOL2. (M, N) Nucleus double immunolabeled for MCAK (green) and BubR1 (red). BubR1 is present as nucleoplasmic aggregates that do not colocalize with MCAK signals. (O, P) Nucleus double immunolabeled for MCAK (green) and CENP-E (red). Both proteins colocalize inside chromocenters. All spermatocytes shown are projections of several focal planes, and are counterstained with DAPI (blue). Scale bar 5 µm.

Interkinesis nuclei are characterized by the presence of a variable number of chromocenters at their internal regions (Figure 5). As occurred during prophase I, the first detectable proteins in interkinesis were the CPC ones. In this sense, we found that INCENP was loaded at chromocenters before Aurora-B (Figure 5A–5D). Afterwards, these two proteins colocalized at the heterochromatic chromocenters (Figure 5C and 5D). Then, we compared the labelings of Aurora-B and SGOL2. SGOL2 was targeted to the centromere after the loading of Aurora-B as during prophase I (Figure 5E–5H). Interestingly, Aurora-B labeled the entire chromocenters, while the SGOL2 signals were inside them (Figure 5G). Following the SGOL2 loading (Figure 5I and 5J), MCAK was detected at the centromeric regions as small spots inside the chromocenters and colocalizing with SGOL2 (Figure 5K and 5L). As during prophase I, we observed that the distribution of the CPC proteins dramatically changed once MCAK was detected at the chromocenters. Thus, they concentrated inside the chromocenters to appear as small spots that colocalize with MCAK and SGOL2 (data not shown). The last proteins loaded to the centromeric region after MCAK were the outer kinetochore proteins BubR1 and CENP-E (Figure 5M–5P). Interestingly, BubR1 appeared only at nucleoli in most interkinesis nuclei, but during the late interkinesis/prophase II transition it relocalized onto kinetochores (Figure S3).

## Discussion

### Loading of CPC Proteins

**Figure 6:**
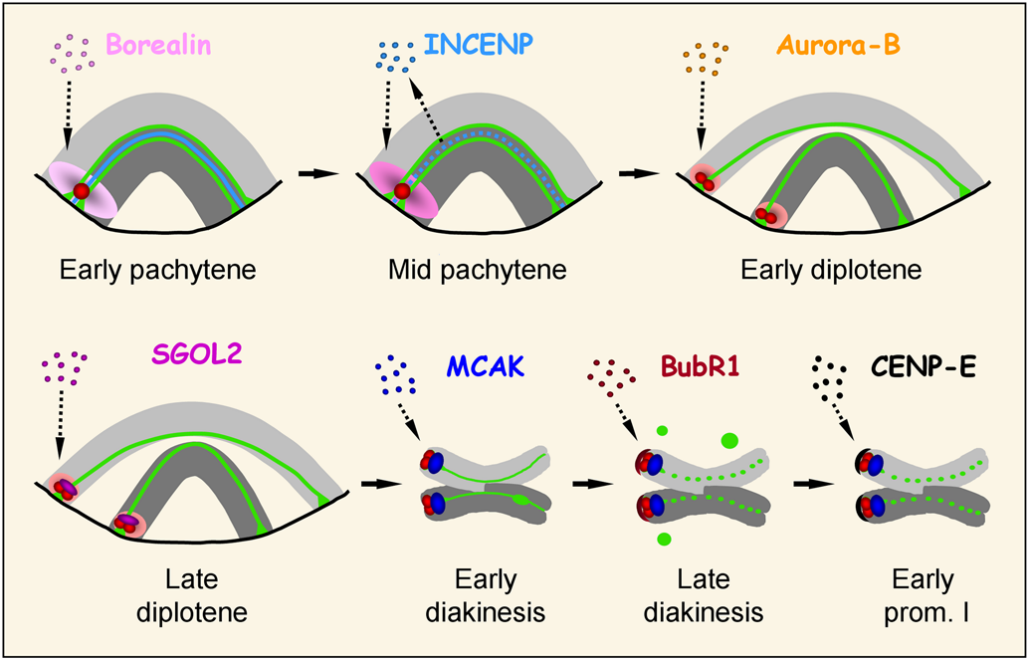
Loading sequence of different inner centromere and outer kinetochore proteins throughout mouse prophase I. A single telocentric bivalent with homologs in light and dark grey is depicted. The kinetochores, as revealed with the ACA serum, are shown in red, and the lateral elements of the synaptonemal complex are shown in green. The bivalent ends are shown associated to the nuclear envelope (single black line) from early pachytene up to late diplotene. Borealin (pink) is the first CPC protein loaded at the pericentromeric heterochromatin, followed by INCENP (light blue) and Aurora-B (orange). Note that INCENP relocalizes from the central element of the synaptonemal complex to the centromere during mid pachytene. After the CPC complex is loaded, SGOL2 (fuchsia) and MCAK (dark blue) are subsequently recruited to the inner centromere. Finally, the outer kinetochore proteins BubR1 (maroon) and CENP-E (black) assemble onto kinetochores. For details see text.

In this study we have analyzed the loading sequence of different inner centromere and outer kinetochore proteins in male mouse meiosis. Our observations lead us to propose a sequence of assembly for those proteins (Figure 6). The first group of proteins that we have detected at the centromeric region, excluding the kinetochoric constitutive ones detected by the ACA serum, are the CPC proteins. During prophase I, these proteins are loaded at the inner centromere between early pachytene and early diplotene. Although it has been previously established that during mitosis the presence of all CPC subunits is necessary for the assembly of the complex at the inner centromere [9], we have detected that during meiosis Borealin, Aurora-B and INCENP are loaded in a precise sequence. Thus, Borealin was the first CPC protein that we found at the heterochromatic chromocenters during early pachytene, followed by INCENP and Aurora-B during mid pachytene and early diplotene, respectively. It has been described that Borealin can bind DNA *in vitro* [31], but it is still unknown if it has any affinity for centromeric DNA. However, Borealin could initiate the sequence of CPC assembly to the inner centromere during meiosis. In this sense, it might bind to the pericentromeric DNA and trigger the loading of the remaining CPC proteins. The next CPC protein in the meiosis assembly sequence, INCENP, presents an N-terminal domain that binds Borealin and Survivin that when depleted prevents their association to the inner centromere [31],[46]. Thus, during meiosis INCENP could also interact with Borealin, and presumably also Survivin, by its N-terminal domain. We have to highlight that INCENP is initially detected at the central element of the synaptonemal complex during zygotene, and relocalizes to the centromeric region by mid pachytene [39]. We do not know whether INCENP may play any specific role at the synaptonemal complex or is just waiting to be assembled at the inner centromere. Finally, the last CPC protein to be loaded at the pericentromeric chromatin is Aurora-B which is described to bind the C-terminal domain of INCENP [33]. This fact suggests that once INCENP is targeted to the inner centromere, Aurora-B would then be loaded.

The sequential loading of the CPC proteins that we have observed during meiosis contrasts with the proposed simultaneous presence of the four CPC proteins for assembling the complex during mitosis [9]. These apparent differences might be due to the long duration of prophase I, in relation to the relatively shorter mitotic one, which allows to accurately analyze the sequential loading of the three CPC proteins. Nevertheless, it is likely that the entire CPC complex is assembled once all the subunits have been accurately loaded.

### Building the Inner Centromere

During male mouse meiosis SGOL2 appears at the inner centromere during late diplotene [19],[41] to protect centromeric cohesin complexes from cleavage by separase during the metaphase I/anaphase I transition [45],[47]. We have found that during late diplotene SGOL2 appears inside the chromocenters where Borealin, INCENP and Aurora-B are present. This result demonstrates that the interaction area of SGOL2 within the centromeric region is smaller than the targeting zone for the CPC proteins. Interestingly, after the SGOL2 loading, the CPC proteins then change their distribution to become restricted to a smaller area that at that time colocalizes with SGOL2. It has been recently proposed that during fission yeast mitosis and budding yeast meiosis Sgo2 and Sgo1 are required, respectively, for the recruitment of some CPC components to the centromere [36],[37],[48]. By contrast, during *Drosophila* meiosis and in *Xenopus* egg extracts, the CPC proteins promote the loading of the single Shugoshin MEI-S322 and xSgo to the inner centromere, respectively [49]. Likewise, in HeLa cells the localization of SGOL2 is dependent on Aurora-B [38]. In this sense, our results indicate that during mouse meiosis the CPC proteins are loaded to centromeric heterochromatin without the participation of SGOL2, but their redistribution from the centromeric heterochromatin to the inner centromere occurs after the loading of SGOL2.

After the loading of SGOL2, and concomitantly with the relocalization of the CPC proteins, we detected the incorporation of MCAK to centromeres. MCAK, Aurora-B and SGOL2 have been involved in the correction of inaccurate merotelic attachments in mitosis [38] and meiosis [19],[41]. Thus, the relocalization of the CPC and the loading of MCAK could involve a reorganization of the centromere in preparation for microtubule interactions. The temporal localization of SGOL2 may indicate its key role in such centromere reorganization, and/or in the recruitment of other inner centromere proteins like MCAK. Indeed, it has been reported that in HeLa cells SGOL2 recruits MCAK to the inner centromere [38]. Our results on *Sgol2^−/−^* spermatocytes support that SGOL2 also recruits MCAK during mouse meiosis since in mutant spermatocytes MCAK never localizes to the inner domain, as occurs in *Sgol2^−/−^* mouse embryonic fibroblasts [45]. In this respect, we have found that in the absence of SGOL2 and MCAK, bivalents align accurately at the metaphase I plate and meiosis progression is not blocked. Consequently, it is uncertain whether MCAK has an essential role during at least meiosis I.

### Outer Kinetochore

We have found that BubR1 is recruited at kinetochores at late diakinesis after the loading of all the studied inner centromere proteins. This is followed by the incorporation of CENP-E onto kinetochores during prometaphase I. These results thus suggest that during mouse meiosis, as occurs in mitosis, the outer kinetochore proteins are loaded on maturing kinetochores once the inner centromere has been completely organized. Indeed, the inhibition of Aurora-B function during mitosis impairs the loading of BubR1, MAD2 and CENP-E to the outer kinetochore [50]. Furthermore, it has been demonstrated that the Aurora-B/INCENP complex induces the localization of MPS1, BUB1, BUB3, and CENP-E to the kinetochores in CSF *Xenopus* egg extracts [51].

The sequence of loading that we found for BubR1 and CENP-E in meiosis seems to be consistent with previous reports in mitosis. Thus, although CENP-E is required to enhance the recruitment and the activity of BubR1 [52], the previous presence of Bub1 and BubR1 is necessary for CENP-E to be properly loaded to the outer kinetochore [26],[27].

### Rebuilding the Centromere for Second Meiotic Division

All the proteins which we have tested in this study are released from the inner centromere and the kinetochore at the end of meiosis I between late anaphase I and the end of telophase I. During this period, INCENP, Aurora-B and CENP-E, relocalize to the spindle midzone and finally disappear [39],[40], and SGOL2, MCAK and BubR1 become undetectable [19],[41]. We have previously shown that SYCP3, a structural component of the lateral elements of the synaptonemal complex, colocalizes with the cohesin subunit RAD21 at the inner domain of metaphase I centromeres. These proteins are released from the inner centromere during interkinesis and are not visualized during meiosis II, thus suggesting that they are not essential for centromere behavior during meiosis II [42]. By contrast, all the proteins analyzed in this study are incorporated again at the centromere during the interkinesis stage with the same loading sequence as during meiosis I. This fact strongly suggests that the structural assembly of the centromere follows a pattern that is conserved in both meiotic divisions. However, important differences may be highlighted, since the assembly of the centromere during meiosis I is initiated during late prophase I, while the assembly for meiosis II takes place during interkinesis. This reveals that the underestimated interkinesis is not just a resting stage between the two meiotic divisions, but a crucial period during mammalian male meiosis, for, at least, chromosome and centromere reorganization for the second meiotic division.

## Materials and Methods

### Squashing of Seminiferous Tubules and Immunofluorescence Microscopy

Testes from adult normal C57BL/6 and *Sgol2^−/−^* [45] male mice were used. All animals were handled in strict accordance with good animal practice as defined by the relevant national and/or local animal welfare bodies, and all animal work was approved by the UAM committee. Testes were removed, detunicated and seminiferous tubules fixed for squashing and subsequent immunofluorescence as previously described [40],[53]. Seminiferous tubules were fixed for 10 min in freshly prepared 2% formaldehyde in PBS (137 mM NaCl, 2.7 mM KCl, 10.1 mM Na_2_HPO_4_, 1.7 mM KH_2_PO_4_, pH 7.4) containing 0.1% Triton X-100 (Sigma). After 5 min, several seminiferous tubules fragments were placed on a slide coated with 1 mg/ml poly-L-lysine (Sigma) with a small drop of fixative, and gently minced with tweezers. The tubules were then squashed and the coverslip removed after freezing in liquid nitrogen. The slides were later rinsed three times for 5 min in PBS, and incubated for 45 min at room temperature or 12 h at 4°C with primary antibodies diluted in PBS. In double labeling experiments, primary antibodies from different host species were incubated simultaneously. Following three washes in PBS for 5 min, the slides were incubated for 30 min at room temperature with secondary antibodies. The slides were subsequently rinsed in PBS and counterstained for 3 min with 5 µg/ml DAPI (4′,6-diamidino-2-phenylindole). After a final rinse in PBS, the slides were mounted with Vectashield (Vector Laboratories) and sealed with nail polish.

Immunofluorescence image stacks were collected on an Olympus BX61 microscope equipped with epifluorescence optics, a motorized z-drive, and an Olympus DP70 digital camera controlled by analySIS software (Soft Imaging System). Stacks were analyzed and processed using the public domain ImageJ software (National Institutes of Health, USA; http://rsb.info.nih.gov/ij). Final images were processed with Adobe Photoshop 7.0 software.

### Antibodies

Kinetochores were detected with a purified human anti-centromere autoantibody (ACA) (Antibodies Incorporated, cat. no. 15-235) at a 1∶50 dilution. Borealin was detected with a rabbit affinity-purified antibody against human Borealin (1647) kindly provided by Dr. W.C. Earnshaw [46] at a 1∶30 dilution. To detect INCENP we used a polyclonal rabbit serum (pAb1186) raised against chicken INCENP kindly provided by Dr. W.C. Earnshaw [54], which also recognizes mouse INCENP [39], at a 1∶100 dilution. Aurora-B kinase was detected with the mouse monoclonal AIM-1 antibody (Transduction Labs) at a 1∶30 dilution. SGOL2 was detected with a rabbit polyclonal serum (K1059) against the C-terminus of mouse SGOL2 kindly provided by Dr. J.L. Barbero [19],[41] at a 1∶20 dilution. To detect MCAK we used affinity-purified sheep and rabbit polyclonal antibodies against human MCAK, kindly provided by Dr. L. Wordeman [18],[55] at 1∶40 and 1∶200 dilutions, respectively. An affinity purified sheep polyclonal antibody against human BubR1 (SBR1.1) kindly provided by Dr. S.S. Taylor [56] was used at a 1∶50 dilution. CENP-E was detected using a polyclonal rabbit serum (pAb1.6) that recognizes the neck region (amino acids 256–817) of human CENP-E kindly provided by Dr. T. Yen [57], at a 1∶100 dilution. Fibrillarin was detected with a human anti-fibrillarin autoantibody (S4) kindly provided by Dr. R. Benavente at a 1∶200 dilution.

The secondary antibodies used were: donkey anti-human IgG (Jackson) at a 1∶150 dilution, donkey anti-rabbit IgG (Jackson) at a 1∶150 dilution, donkey anti-mouse IgG (Jackson) at a 1∶150 dilution, donkey anti-sheep IgG (Jackson) at a 1∶40 dilution. All of them were conjugated with either Texas Red or fluorescein isothiocyanate (FITC).

## Supporting Information

Figure S1 Distribution of BubR1 during meiosis I. Spermatocytes are double immunolabeled for BubR1 (green) and kinetochores (ACA, red). (A–D) Zygotene and pachytene spermatocytes. BubR1 only labels the nucleoli (arrowheads). (E–H) Diplotene and early diakinesis spermatocytes. BubR1 appears at nucleoli (arrowheads) and as small nucleoplasmic aggregates (arrows) that do not colocalize with kinetochores. (I–L) Late diakinesis and prometaphase I spermatocytes. BubR1 appears at the kinetochores, and during late diakinesis is still present at the disintegrating nucleolus (arrowhead). (M, N) Prometaphase I spermatocyte. BubR1 appears enriched at the kinetochores (arrows) of an unaligned bivalent. (O–T) Metaphase I, anaphase I, and telophase I spermatocytes. BubR1 has mostly disappeared from kinetochores. All spermatocytes shown are projections of several focal planes, and are counterstained with DAPI (blue). Scale bar 10 µm.
(5.18 MB TIF)

Figure S2 Distribution of BubR1 at nucleoli during prophase I. Spermatocytes are double immunolabeled for BubR1 (green) and fibrillarin (red). (A, B) Pachytene spermatocyte. BubR1 colocalizes with fibrillarin at nucleoli (arrowheads), but not at the single Cajal body (CB). (C, D) Early diakinesis nucleus. BubR1 appears at a single nucleolar remnant (arrowhead) colocalizing with fibrillarin, and at numerous aggregates (arrows) in the nucleoplasm. The spermatocytes shown are projections of several focal planes, and are counterstained with DAPI (blue). Scale bar 10 µm.
(0.88 MB TIF)

Figure S3 Distribution of BubR1 during meiosis II. Spermatocytes and an early spermatid are double immunolabeled for BubR1 (green) and kinetochores (ACA, red). (A, B) Interkinesis spermatocyte. BubR1 labels nucleoli (arrowheads). (C, D) Prophase II spermatocyte. BubR1 is located at the disintegrating nucleolar masses (arrowhead) and at kinetochores. (E, F) Prometaphase II spermatocyte. BubR1 is enriched at the kinetochores of unaligned chromosomes. (G–L) Metaphase II, anaphase II, and telophase II spermatocytes. BubR1 has mostly disappeared from kinetochores. (M, N) Early round spermatid. BubR1 is only detected at the nucleolus (arrowhead). All spermatocytes shown are projections of several focal planes, and are counterstained with DAPI (blue). Scale bar 10 µm.
(2.87 MB TIF)
