# Supplementary figures and images for: Sequential Assembly of Centromeric Proteins in Male Mouse Meiosis
Source: PLoS Genet. 2009 Mar 13;5(3):e1000417. doi: 10.1371/journal.pgen.1000417 (PMC2652116; doi:10.1371/journal.pgen.1000417)

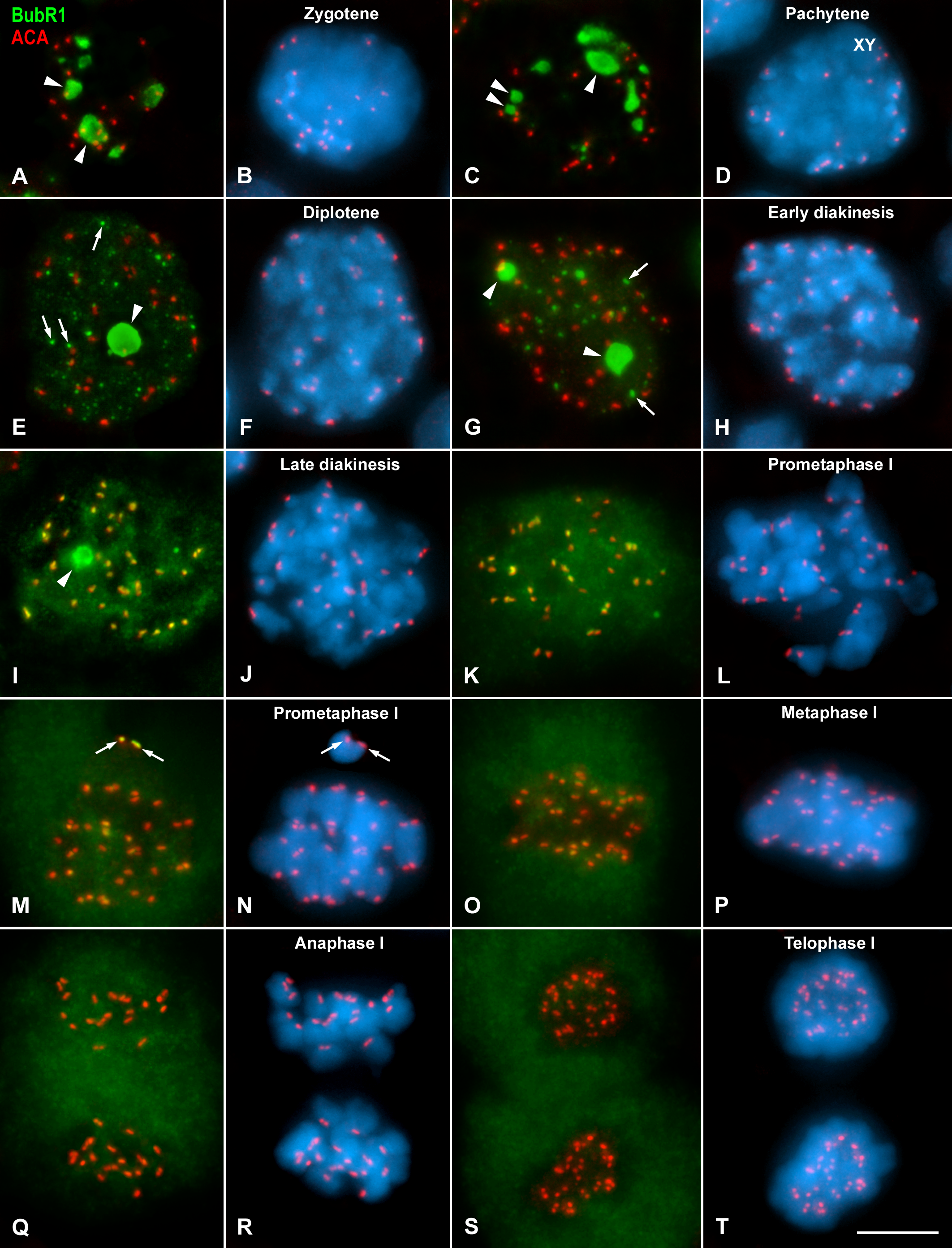

Supplement: Figure S1 — Distribution of BubR1 during meiosis I. Spermatocytes are double immunolabeled for BubR1 (green) and kinetochores (ACA, red). (A–D) Zygotene and pachytene spermatocytes. BubR1 only labels the nucleoli (arrowheads). (E–H) Diplotene and early diakinesis spermatocytes. BubR1 appears at nucleoli (arrowheads) and as small nucleoplasmic aggregates (arrows) that do not colocalize with kinetochores. (I–L) Late diakinesis and prometaphase I spermatocytes. BubR1 appears at the kinetochores, and during late diakinesis is still present at the disintegrating nucleolus (arrowhead). (M, N) Prometaphase I spermatocyte. BubR1 appears enriched at the kinetochores (arrows) of an unaligned bivalent. (O–T) Metaphase I, anaphase I, and telophase I spermatocytes. BubR1 has mostly disappeared from kinetochores. All spermatocytes shown are projections of several focal planes, and are counterstained with DAPI (blue). Scale bar 10 µm. (5.18 MB TIF) [file pgen.1000417.s001.tif]

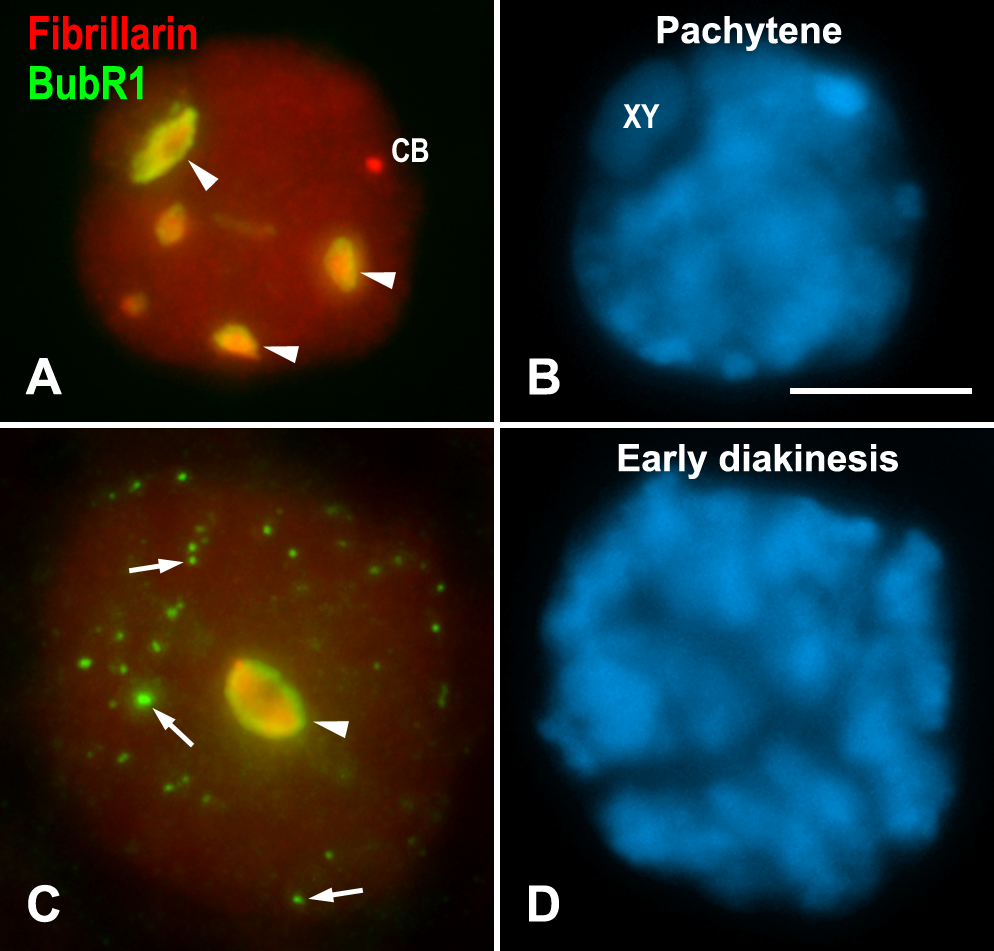

Supplement: Figure S2 — Distribution of BubR1 at nucleoli during prophase I. Spermatocytes are double immunolabeled for BubR1 (green) and fibrillarin (red). (A, B) Pachytene spermatocyte. BubR1 colocalizes with fibrillarin at nucleoli (arrowheads), but not at the single Cajal body (CB). (C, D) Early diakinesis nucleus. BubR1 appears at a single nucleolar remnant (arrowhead) colocalizing with fibrillarin, and at numerous aggregates (arrows) in the nucleoplasm. The spermatocytes shown are projections of several focal planes, and are counterstained with DAPI (blue). Scale bar 10 µm. (0.88 MB TIF) [file pgen.1000417.s002.tif]

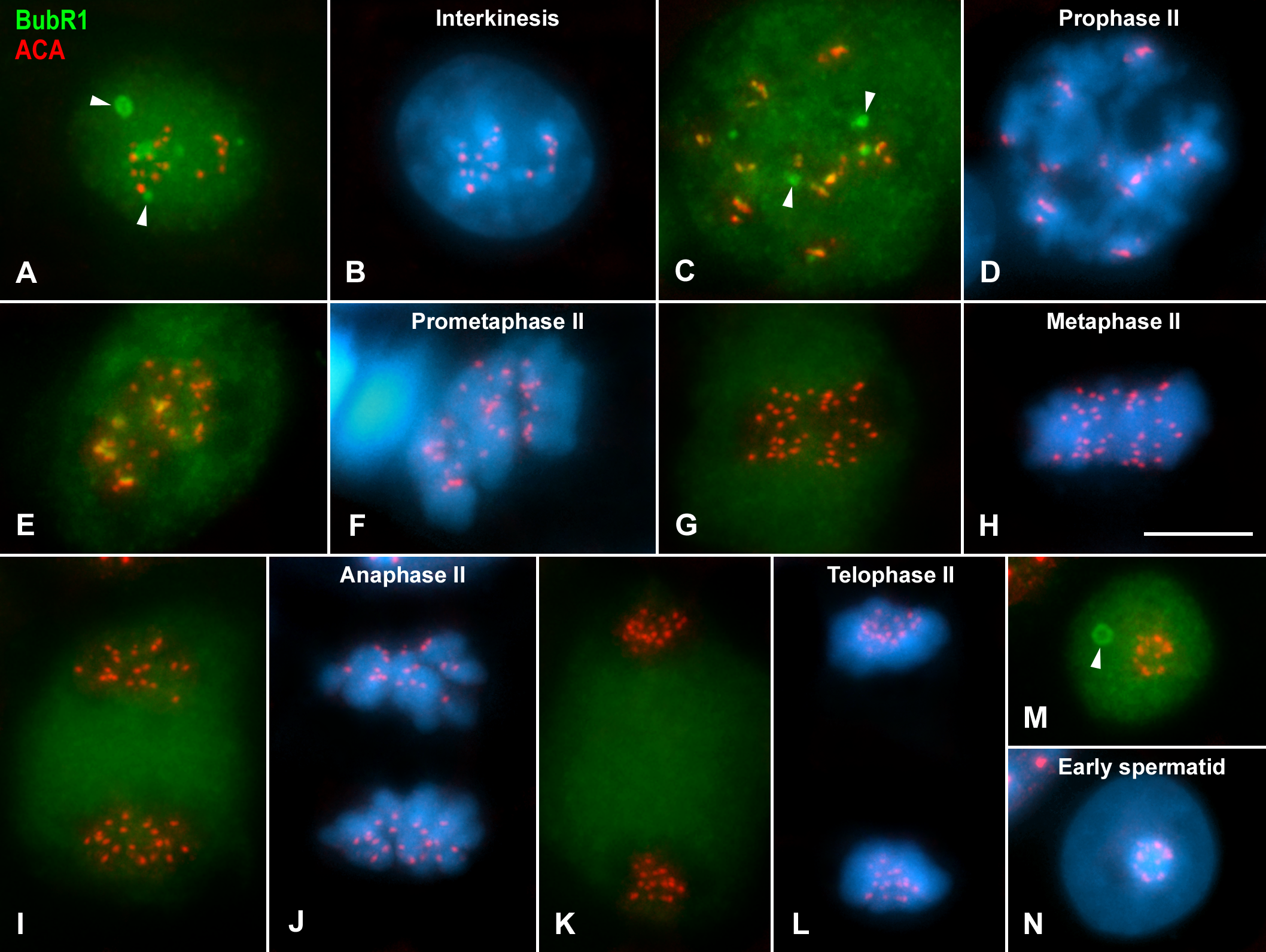

Supplement: Figure S3 — Distribution of BubR1 during meiosis II. Spermatocytes and an early spermatid are double immunolabeled for BubR1 (green) and kinetochores (ACA, red). (A, B) Interkinesis spermatocyte. BubR1 labels nucleoli (arrowheads). (C, D) Prophase II spermatocyte. BubR1 is located at the disintegrating nucleolar masses (arrowhead) and at kinetochores. (E, F) Prometaphase II spermatocyte. BubR1 is enriched at the kinetochores of unaligned chromosomes. (G–L) Metaphase II, anaphase II, and telophase II spermatocytes. BubR1 has mostly disappeared from kinetochores. (M, N) Early round spermatid. BubR1 is only detected at the nucleolus (arrowhead). All spermatocytes shown are projections of several focal planes, and are counterstained with DAPI (blue). Scale bar 10 µm. (2.87 MB TIF) [file pgen.1000417.s003.tif]
